# Protective effects of *Spirulina maxima *on hyperlipidemia and oxidative-stress induced by lead acetate in the liver and kidney

**DOI:** 10.1186/1476-511X-9-35

**Published:** 2010-03-31

**Authors:** Johny C Ponce-Canchihuamán, Oscar Pérez-Méndez, Rolando Hernández-Muñoz, Patricia V Torres-Durán, Marco A Juárez-Oropeza

**Affiliations:** 1 Departamento de Bioquímica, Facultad de Medicina, Universidad Nacional Autónoma de México. A.P. 70-159, México, D.F. 04510, México; 2 Departamento de Biología Molecular, Instituto Nacional de Cardiología, Ignacio Chávez, México; 3 Departamento de Biología Celular, Instituto de Fisiología Celular, Universidad Nacional Autónoma de México, México

## Abstract

**Background:**

Oxidative damage has been proposed as a possible mechanism involved in lead toxicity, specially affecting the liver and kidney. Previous studies have shown the antioxidant effect of *Spirulina maxima *in several experimental models of oxidative stress. The current study was carried out to evaluate the antioxidant activity of *Spirulina maxima *against lead acetate-induced hyperlipidemia and oxidative damage in the liver and kidney of male rats. Control animals were fed on a standard diet and did not receive lead acetate (Control group). Experimental animals were fed on a standard laboratory diet with or without *Spirulina maxima *5% in the standard laboratory diet and treated with three doses of lead acetate (25 mg each/weekly, intraperitoneal injection) (lead acetate with *Spirulina*, and lead acetate without *Spirulina *groups).

**Results:**

The results showed that *Spirulina maxima *prevented the lead acetate-induced significant changes on plasma and liver lipid levels and on the antioxidant status of the liver and kidney. On the other hand, *Spirulina maxima *succeeded to improve the biochemical parameters of the liver and kidney towards the normal values of the Control group.

**Conclusions:**

It was concluded that *Spirulina maxima *has protective effects on lead acetate-induced damage, and that the effects are associated with the antioxidant effect of *Spirulina*.

## Background

The lead is a xenobiotic, persistent toxic [1,2], as other xenobiotics induces to different health risks since the fetal stage until senescence. On the other hand, although lead is one of the most useful metals, it is also one of the most toxic ones [3]. Also, both occupational and environmental exposures remain a serious problem in many developing and industrializing countries [4].

Several reports have indicated that lead can cause neurological, hematological, gastrointestinal, reproductive, circulatory, and immunological pathologies, all of them related to the dose and the amount of time of lead exposure [5-8]. Also, the health risks occasioned by exposure to lead are considered public health problems at a world level.

The liver plays a major role in lead's metabolism, and it is in special risk due to the oxidative action of this xenobiotic; given the unquestionable evidence that lead-induced lipid peroxidation of cellular membranes, plays a crucial role in the mechanisms of hepatotoxic action of these xenobiotics [9]. On the other hand, lead is known to also affect the kidney, which is another important target [10]. Lead produces oxidative damage in the kidney as evidenced by enhancing lipid peroxidation (LIP) [11,12].

*In vivo *and *in vitro *studies suggest that lipid metabolism is altered both in acute and chronic exposure to lead [13]. Lead inhibits antioxidant enzyme activity, such as superoxide dismutase and catalase, and also decreases the level of glutathione, increasing lipid peroxidation [14,15], which harms proteins, cell membranes and DNA, among others. However, this damage could decline when antioxidants such as flaxseed are supplied [16].

The cyanobacterium *Spirulina maxima *or *Arthrospira maxima *has shown hepatoprotective effects in rats, and other experimental models [17]. Also, it has been demonstrated hypolipemiant and antioxidant effects of the *Spirulina *in humans [18,19]. In addition, *Spirulina *is a source of β-carotene, α-tocopherol, and phycocyanin, molecules with antioxidant properties [20-22].

Heavy metal poisoning like lead cause adverse effects to hepatic cells because after lead exposure, liver is one of the major organs involved in the storage, biotransformation and detoxification. Lead also, affects the kidney, which is another important organ that participates in the detoxification. The aim of the current study was to evaluate the effects of *Spirulina maxima *on lipid metabolism and the antioxidant system in the hepato and nephrotoxicity induced by lead.

## Materials and methods

### Reagents, chemicals and Spirulina

All reagents and chemicals used were of analytical grade. Lead acetate (LA) was purchased from Sigma Chemical Co. (St. Louis, MO, USA). Powdered *Spirulina maxima *used in the experimental diet was purchased from Alimentos Esenciales para la Humanidad (Mexico) and was free of lead. Total cholesterol (TC), triacylglycerols (TAG) and alanine aminotransferase (ALT) were assessed by enzymatic kits (Spinreact, Mexico). Total lipids (TL) of the liver were extracted with 20 volumes of chloroform/methanol (3:1, v/v; Merck, Mexico).

### Experimental animals

Two-month old Wistar male rats (180-200 g) were maintained and housed in a room with a controlled temperature (12-15°C), artificially illuminated with dark-light cycles (07:00 to 19:00 h as light). After an acclimatization period of one week, the animals were divided at random into three groups (6 rats/group) and housed in filter-top polycarbonate cages. All the animals received humane care in compliance with the guidelines of Animal Care [23].

After lead exposure, the animals were observed inter-daily for signs of toxicity. Body weight was recorded inter-daily during the experimental period. The standard laboratory food for rodents (Purina, Mexico) with or without *Spirulina *5% was used to feed all the animals (diets *Spirulina *and without-*Spirulina*, respectively). The amount of diet provided was 20 g/day/rat, water was supplied *ad libitum*. Lead acetate treatment was given to the experimental groups, three times (25 mg/rat, weekly). Isotonic saline was used as a vehicle (0.5 mL, i.p.), and given to the Control group as for the experimental groups.

### Experimental design

Animals within different treatment groups were maintained on their respective diets, followed for 30 days and received the dose of LA the days 14, 21, and 28 as follows: Group 1 (untreated, Control) fed on Purina diet and three doses of isotonic saline, i.p. Group 2 (LA treated, LA without *Spirulina*: LAwS) fed on Purina diet and three doses of 25 mg of lead acetate, i.p. Group 3 (LA treated, LA *Spirulina*: LAS) fed on *Spirulina *diet and three doses of 25 mg of lead acetate, i.p.

At the end of the experimental period of 30 days, the rats were deprived of food overnight by 12 hours and all the animals were killed by cervical dislocation at 24 h post-treatment, after being anaesthetized in a gas ether atmosphere. Blood was collected by exsanguination in heparinized test tubes. Plasma was separated by centrifugation and stored at -78°C until lipid analyses were performed. The ALT analyses were performed using fresh plasma. The liver and the kidney were finely excised and weighed. A segment of 1 g fresh liver was removed for total lipids analyses. The enzyme activity of superoxide dismutase (SOD) and catalase (CAT), as well as the reduced glutathione (GSH) and thiobarbituric acid-reactive substances (TBARS) levels were assessed with fresh liver homogenate and fresh kidney homogenate. The TBARS levels were reported in concentrations of malondialdehyde (MDA).

### Lipid analyses

Determinations of TC and TAG were made using commercial colorimetric enzymatic methods, following the recommendations by the providers.

For the liver and the kidney samples, 1 g of tissue was homogenized in phosphate buffer; and the total lipids were extracted with chloroform/methanol mixture by a modified Folch's method [24], and were determined gravimetrically.

### Alanine aminotransferase analysis

The level of ALT was assessed using commercial method at 340 nm.

### Antioxidant status indicators

The enzyme activity of SOD [25], and CAT [26], as well as the GSH levels [27] were assessed in the liver and kidney homogenates. Lipid peroxides from the liver and kidney homogenates were measured by the determining of TBARS formation [28].

The activity of SOD was determined by the Kono's method [25], through the capability of inhibition of NBT (nitroblue of tetrazolium) reduction. The changes in the absorbance were recorder at 560 nm during five minutes.

The activity of CAT was determined by the Aebi's method [26], through the disappearance of H_2_O_2_. The changes in the absorbance were determined at 240 nm during five minutes.

The level of GSH was evaluated by the method of Owens [27]; previously, the sample homogenates were mixed with 28% trichloroacetic acid (TCA, w/v) in order to discard the protein fraction. The supernatant content of GSH was performer using 5,5'-dithiobis (2-nitrobenzoic acid) at 412 nm.

Products of lipid peroxidation were determined by the Ohkawa's method [28]; briefly, the free protein supernatant fraction was used to evaluate the TBARS production [1]. The absorbance of the resulting chromophore was determined at 535 nm, and expressed as MDA production, using 1,1,3,3-tetraethoxypropane as standard.

Total protein content was assessed using Bradford's method [29], using albumin as standard at 595 nm.

The above-mentioned endogenous antioxidant indicators were evaluated using a Genesis UV10 spectrophotometer (Thermo Electron Co, USA), and their values were expressed by protein content.

### Statistical analysis

Results were evaluated statistically using one-way analyses of variance (ANOVA) with Bonferroni test of the Statistical Package for the Social Sciences (SPSS, v. 16). The significance of the differences among treatment groups, to all statements of significance were based on the probability of p < 0.05.

## Results

### Body weight of rats

The effect of *Spirulina maxima *(Sm) and lead acetate on animal body weight gain in the different treatments revealed that lead acetate (LAwS) alone significantly decreased the body weight (p < 0.05). Then, at the end of the experiment, body weight gain in the LAS group (50.94 ± 5.23 g) was similar to the Control group (52.36 ± 4.51 g), but the LAwS group (47.41 ± 3.83 g) gained less weight than the Control group (p < 0.05).

### Determination of lipids in plasma and liver

As shown in Table 1, in both plasma and liver, TC and TAG levels were higher in the LAwS group compared to the Control group (p < 0.05). The LAS group showed similar results to the Control group but significantly lower TC and TAG levels than the LAwS group (p < 0.05). The total lipids in liver were similar levels among the three groups.

**Table 1:** Effects of *Spirulina maxima *on biochemical parameters during sub-chronic lead exposure in rats.

	Experimental Groups		
	
Variable	Control	LAwS	LAS
***Aminotransferase***			
Alanine aminotransferase (ALT) (U/L)^a^	28.76 ± 5.84	46.51 ± 4.04^b^	31.72 ± 4.72^c^
***Plasma Lipids ****(mg/dL)*			
Total Cholesterol (TC)^a^	80.07 ± 2.54	107.83 ± 2.69^b^	82.99 ± 12.70^c^
Triacylglycerols (TAG)^a^	91.13 ± 2.45	125.24 ± 4.43^b^	94.35 ± 5.09^c^
***Liver Lipids ****(μg/mgLip)*			
Total Cholesterol (CT)^a^	133.49 ± 10.73	174.18 ± 13.13^b^	134.32 ± 10.76^c^
Triacylglycerols (TAG)^a^	65.05 ± 6.84	197.93 ± 9.05^b^	70.25 ± 11.22^c^
Total Lipids (TL) (mgLip/g Liver)	48.98 ± 2.38	52.47 ± 3.27	49.40 ± 3.28

Values are mean ± SD (n = 6); in each group. LA (Lead acetate, 75 mg). Control (no LA and without *Spirulina*), LAwS (LA without *Spirulina*); LAS (LA with *Spirulina*)

^a^p < 0.05, Significantly different. ANOVA.

^b^p < 0.05, LAwS group compared with the Control group. Bonferroni test.

^c^p < 0.05, LAwS group compared with the LAS group. Bonferroni test.

### Evaluation of ALT activity

The LAwS group showed higher ALT activity than the Control (p < 0.05) and LAS (p < 0.05) groups. On the other hand, the LAS group presented similar activity to the Control group (Table 1).

### Evaluation of the antioxidant status indicators

**Figure 1:**
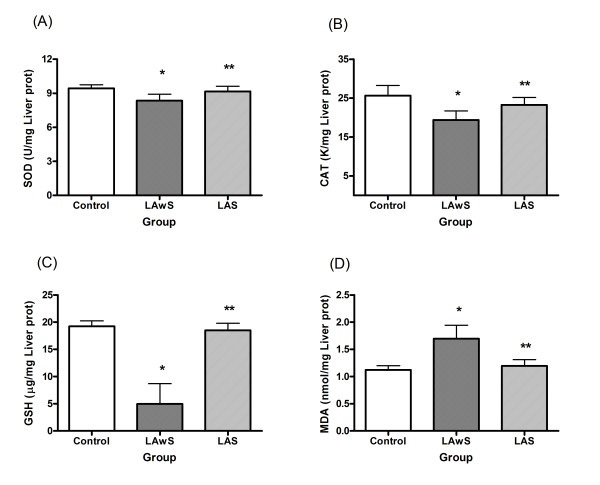
**Effects of *Spirulina maxima *on liver oxidative status indicators during lead exposure in rats**. The animals were treated with a vehicle or with 75 mg of lead acetate (LA, 25 mg/0.5 ml isotonic saline each, i.p., and three times on days 14, 21 and 28, and sacrificed on day 30). Values are expressed in mean ± SD of n = 6 rats. (A). The total Superoxide Dismutase (SOD) activity. (B). The Catalase (CAT) activity. (C). The Glutathione (GSH) levels. (D). The Thiobarbituric Acid-Reactive Substances (TBARS) levels. Control (no LA and without *Spirulina*), LAwS (LA without *Spirulina*), LAS (LA with *Spirulina*). *p < 0.05, LAwS group compared with the Control group. ANOVA with Bonferroni test. **p < 0.05, LAwS group compared with the LAS group. ANOVA with Bonferroni test.

In the liver:

It was found that the enzyme activities of SOD and CAT in rat liver were lower in LAwS group compared to the Control (p < 0.05). The LAS group showed similar activities compared to the Control group but significantly higher activities than the LAwS group (p < 0.05) (Fig. 1).

The level of GSH in the liver was lower in the LAwS group compared to the Control (p < 0.05) and the LAS (p < 0.05) groups. The LAS group presented similar GSH levels compared to the Control group (Fig. 1).

The level of MDA in rat liver was 1.51 and 1.42 times higher in the LAwS group than the Control (p < 0.05) and LAS (p < 0.05) groups, respectively. The LAS group presented a similar MDA level compared to the Control group (Fig. 1).

**Figure 2:**
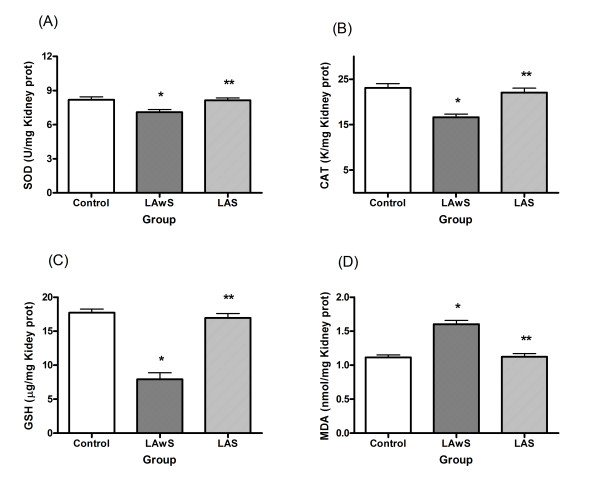
**Effects of *Spirulina maxima *on kidney oxidative status indicators during lead exposure in rats**. The animals were treated with a vehicle or with 75 mg of lead acetate (LA, 25 mg/0.5 ml isotonic saline each, i.p., and three times on days 14, 21 and 28, and sacrificed on day 30). Values are expressed in mean ± SD of n = 6 rats. (A). The total Superoxide Dismutase (SOD) activity. (B). The Catalase (CAT) activity. (C). The Glutathione (GSH) levels. (D). The Thiobarbituric Acid-Reactive Substances (TBARS) levels. Control (no LA and without *Spirulina*), LAwS (LA without *Spirulina*), LAS (LA with *Spirulina*). *p < 0.05, LAwS group compared with the Control group. ANOVA with Bonferroni test. **p < 0.05, LAwS group compared with the LAS group. ANOVA with Bonferroni test.

In the kidney:

It was found that the enzyme activities of SOD and CAT in rat kidney were decreased in LAwS group compared to the Control group (p < 0.05). The LAS group showed similar activity of SOD and CAT compared to the Control group, and was significantly different compared to the LAwS group (p < 0.05) (Fig. 2).

The level of GSH in kidney was lower in LAwS group compared to the Control (p < 0.05) and LAS (p < 0.05) groups. The LAS group presented a similar GSH level compared to the Control group (Fig. 2).

The level of MDA in the rat kidney was 1.44 and 1.43 folds higher in the LAwS group compared to the Control (p < 0.05) and LAS (p < 0.05) groups, respectively. The LAS group presented a similar MDA level compared to the Control group (Fig. 2).

## Discussion

In the present study, the role of Sm on hyperlipidemia and oxidative damage in LA toxicity of liver and kidney was investigated in male Wistar rats. The dose of LA was based on literature reports [30] and on preliminary studies. The lead exposure was of the sub chronic type. The i.p. route was chosen because it is less stressful to rats, and also because the concentration of lead in the blood could reach levels considered toxic to humans [31]. The dose of Sm was based on previous work [32]. The results indicated that the animals treated with LA (LAwS) showed a decrease in body weight gain; similar observations were reported by El-Nekeety et al [11]. Also, it was found that the mean body weight of the animals treated with LA (LAwS) was lower compared to the Control and LAS groups.

The liver is considered one of the target organs affected by lead toxicity owing to its storage in the liver after lead exposure. Also, the liver being one of the major organs involved in the storage, biotransformation and detoxification of toxic substances, is of interest in heavy metal poisoning [33]. The activity of ALT is one of the indicators of hepatotoxicity [34]; in the present study, treatment with LA (LAwS) induced a slight but significant increase of ALT compared to the other groups. On the other hand, the results also showed that the LA significantly increased plasma levels of CT and TAG in the LAwS group compared with the other groups. The increased levels of lipids with increased levels of ALT may indicate liver dysfunction [35]. These results showed that the exposure to lead affects hepatic tissue, which is consistent with other reports [36,37].

Lead is a heavy metal that produces oxidative damage in the liver by enhancing lipid peroxidation (LIP) [11]. Lead toxicity leads to free radical damage by two separate, although related, pathways: (1) the generation of reactive oxygen species (ROS), including hydroperoxides, singlet oxygen, and hydrogen peroxides, evaluated by MDA levels as the final products of LIP, and (2) the direct depletion of antioxidant reserves [16]. In the present study, treatment with LA (LAwS) resulted in a significant increase of LIP as indicated by the significant increase of MDA levels and the significant decrease of GSH levels. Our results are in agreement with other previous studies [16,2,38]. The presence of LIP observed in the current study was also due to decreased SOD and CAT activities, both indicators of oxidative stress [16]. The possible explanation could be related to the proposed role of GSH in the active excretion of lead through bile by binding to the thiol group of GSH and then being excreted. A decrease in GSH levels could lead to oxidative stress and a consequent increase in LIP [2,11].

Lead is known to adversely affect many organs, where the kidney is another of the important targets [10]. Lead produces oxidative damage in the kidney, by enhancing LIP [11,12]. In the present study, treatment with LA (LAwS) resulted in a significant increase of LIP as indicated by the significant increase of MDA levels and the significant decrease of GSH levels. Similar results have been reported [12,38-40]. The observed LIP in the current study may also assume that there was a disruption of prooxidant/antioxidant balance in lead exposure. However, the source of prooxidant formation during lead-induced oxidative stress has not been extensively studied [10,41]. In addition, it has been reported that the δ-aminolevulinic acid dehydratase (ALAD) inhibition may result in the accumulation of ALA (aminolevulinic acid), and accumulated ALA has been involved in lead-induced oxidative damage by causing formation of reactive oxygen species [42]. It is reasonable to speculate that the increased LIP found in the present study was triggered by ALA accumulation resulting from lead-induced ALAD inhibition.

Lead also induces oxidative damage to the membranes by the accumulation of oxidant metabolites (such as ALA, free protoporphyrins, heme and iron ions) and by direct or indirect inhibition of antioxidant enzymes, reducing the total antioxidant protection of the cell, affecting membrane structure and function and altering physiological processes of organs and tissues [43]. These damages are reflected in cellular structural changes and explain the close relationship between the morphological changes found in the kidneys of lead exposed animals with the molecular and physiological changes with respect to the TBARS levels showed by Navarro-Moreno et al [40].

The present results clearly indicate that the informed biochemical determinations (metabolism of lipids and antioxidant system) [2,11,35] provide evidence of lead toxicity in liver and kidney, and also, the protective effects of Sm as reported in other studies [17,19]. On the other hand, the Sm does not induce any harmful effects on the animals [44,45]. Rather, Sm succeeded to induce an improvement in body weight and the biochemical parameters. Several reports have indicated that Sm has antihyperlipemic [17,18,46] and antioxidant [19,46,47] effects due to its higher content of some macro- and micronutrients including high quality protein, iron, gamma-linolenic fatty acid, carotenoids, vitamins B1 and B2 [48] β-carotene, α-tocopherol and phycocyanin [20]. The phycocyanin has been considered the predominant compound in the antioxidant activity of the *Spirulina *[21,22].

In this study, co-treatment of LA and Sm (LAS) resulted in a significant improvement of all biochemical parameters tested of the plasma, liver, and kidney. Therefore, this cyanobaterium may play a protective role against LA-mediated liver and kidney injury in sub-chronic exposure. These results demonstrated that Sm has antihyperlipemic and antioxidant properties.

## Conclusions

This study has demonstrated that exposure to lead could have generated oxidative stress which resulted in the elevation of lipids both in plasma and liver, as well as lipid peroxidation in the liver and kidney associated with the reduction in the antioxidant status. *Spirulina maxima *co-treatment resulted in the prevention of the lead-induced damages. The protective effects of *Spirulina maxima *may be due to the radical scavenging activity of its components. Consequently, *Spirulina maxima *could be useful in the preventive treatment of lead toxicity.

## Competing interests

The authors declare that they have no competing interests.

## Authors' contributions

JCPC contributed to the design, experimental work, analysis and discussion of the results, and the writing of the manuscript. OPM participated in the interpretation and review of the data. RHM participated in the interpretation and review of the data. PVTD participated in the interpretation and review of the data. MAJO participated in the design of the study, review of the manuscript and discussion of the results and providing funding for the study. All authors have read and approved this manuscript.
